# Recent trends in opioid prescriptions in Korea from 2002 to 2015 based on the Korean NHIS-NSC cohort

**DOI:** 10.4178/epih.e2022029

**Published:** 2022-02-21

**Authors:** Joungyoun Kim, Sang-Jun Shin, Jihyun Yoon, Hyeong-Seop Kim, Jae-woo Lee, Ye-seul Kim, Yonghwan Kim, Hyo-Sun You, Hee-Taik Kang

**Affiliations:** 1 College of Nursing, Mo-Im Kim Nursing Research Institute, Yonsei University, Seoul, Korea; 2 Department of Information & Statistics, Chungbuk National University, Cheongju, Korea; 3 Department of Family Medicine, Yonsei University College of Medicine, Yongin, Korea; 4 Clinical Trials Center, Severance Hospital, Yonsei University Health System, Seoul, Korea; 5 Department of Family Medicine, Chungbuk National University Hospital, Cheongju, Korea; 6 Department of Family Medicine, Chungbuk National University College of Medicine, Cheongju, Korea

**Keywords:** Analgesics, Opioid, Fentanyl, Hydromorphone, Morphine, Oxycodone

## Abstract

**OBJECTIVES:**

Opioids are prescribed to treat moderate to severe pain. We investigated recent trends in opioid (morphine, oxycodone, fentanyl, and hydromorphone) prescriptions using data from the Korean National Health Insurance Service-National Sample Cohort between 2002 and 2015.

**METHODS:**

The morphine milligram equivalent (MME) was calculated to standardize the relative potency of opioids. The number (cases) or amount (MME) of annual opioid prescriptions per 10,000 registrants was computed to analyze trends in opioid prescriptions after age standardization. Joinpoint regression analysis was conducted to calculate the annual percentage change and average annual percentage change (AAPC).

**RESULTS:**

The number (cases) of prescriptions per 10,000 registrants increased from 0.07 in 2002 to 41.23 in 2015 (AAPC, 76.0%; 95% confidence interval [CI], 61.6 to 91.7). The MME per 10,000 registrants increased from 15.06 in 2002 to 40,727.80 in 2015 (AAPC, 103.0%; 95% CI, 78.2 to 131.3). The highest AAPC of prescriptions and MME per 10,000 registrants were observed in the elderly (60-69 years) and in patients treated at general hospitals. Fentanyl prescriptions increased most rapidly among the 4 opioids.

**CONCLUSIONS:**

Consumption of opioids greatly increased in Korea over the 14-year study period.

## GRAPHICAL ABSTRACT

[Fig f3-epih-44-e2022029]

## INTRODUCTION

The International Association for the Study of Pain defines pain as an unpleasant sensory or emotional experience associated with actual or potential tissue damage or described in terms of such damage [1]. Pain, also known as the fifth vital sign, has a significant impact on patients’ prognoses and quality of life [2]. According to a systematic review in 2016 [3], approximately 30-50% of cancer patients experienced moderate to severe pain, and the proportion of patients with pain increased in those with more advanced disease. Another study found that the prevalence of moderate to severe pain among all cancer patients was more than 80%, and approximately 60% of patients felt that their pain was adequately managed [4]. The prevalence of chronic non-cancer pain varies by country, with the United States reporting 30% and European countries 19% [5,6].

According to the 3-step analgesic ladder to relieve cancer pain proposed by the World Health Organization (WHO), opioids are very important drugs for the treatment of moderate to severe pain [7]. Recent pain management guidelines recommend that doctors prescribe analgesics such as opioids depending on patients’ symptom severity [8]. The utilization of opioids has markedly increased in many Asian countries, including Taiwan, Malaysia, and Singapore, in addition to Western countries such as the United States, Canada, and European countries [9-12]. However, there is a lack of studies demonstrating trends in prescriptions of strong opioids in Korea.

Therefore, the purpose of this descriptive study was to provide data on recent trends in opioid prescriptions in terms of cases (prescription numbers) and amounts (morphine milligram equivalent; MME) in Korea over 14 years (from 2002 to 2015) using data from the Korean National Health Insurance Service (NHIS)-National Sample Cohort (NSC).

## MATERIALS AND METHODS

### Study population and total prescriptions

This study was analyzed adults older than 20 years in the NHIS-NSC between 2002 and 2015. Most Koreans subscribe to the obligatory universal national health insurance provided by the Korean NHIS. The sample size of the NHIS-NSC database is approximately 1 million, comprising 2% of randomly selected Koreans who had maintained the qualifications for at least 1 year as of December 2006. To secure representation of the Korean population, stratified sampling was performed considering sex, age, income level, and region. The registered subjects were followed from January 1, 2002 to December 31, 2015. The NHIS-NSC data provide subjects’ medical history records, including diagnosis codes, prescription details, and health screening results between 2002 and 2015. In addition, the cohort data include socio-demographic information, such as age, sex, death, past medical history (e.g., malignant neoplasms, hypertension, and diabetes), health behaviors (e.g., physical activity, cigarette smoking, and alcohol intake), monthly household income, and information from the healthcare units that patients visited. A detailed explanation of the NHIS-NCS has been published previously [13]. Because the year of qualification was 2006, no deaths were observed until 2006. To maintain the age structure over time, approximately 9,000 newborns have been added to the dataset every year since 2006.

### Definition and variables

The opioids considered in our analysis were morphine, oxycodone, fentanyl, and hydromorphone. The primary interests were trends in the number (or amount) of annual opioid prescriptions during the study period, as follows: (1) the number of annual opioid prescriptions (cases) per 10,000 people and (2) the amount of annual opioid prescriptions per 10,000 registrants expressed as MME. The MME of each opioid reflects its analgesic potency relative to morphine. Based on the prescription information of NHIS-NSC data, MME was computed as “strength per unit× (daily dose count× 1 dose)× MME conversion factor” to standardize the potency across opioids or dose formulations (e.g., tablet or patch). MME conversion factors were obtained from the literature [14]. In addition, the annual trends in opioid prescriptions were investigated in subgroups defined by age, type of medical institution, and opioid type. Medical institutions were categorized into (1) general hospitals (or medical institutions with more than 100 beds and several specialized departments as designated by law); (2) hospitals (medical institutions with 30 to 99 beds); and (3) clinics (medical institutions with fewer than 30 beds).

### Statistical analysis

The annual number (or amount) of opioids per 10,000 registrants was calculated by dividing the total number (or amount) of opioid prescriptions in a year by the number of registered people at the beginning of the study year and multiplying by 10,000. To account for the changing age structure of the data, we applied agestandardization in each year to the standard population, which was the 2002 Korean population structure. Furthermore, joinpoint regression analysis was performed to detect significant changes [15]. The overall trend from 2002 to 2015 was calculated as the average annual percentage change (AAPC). When the trend changed significantly, trends in shorter time segments were calculated as the annual percentage change (APC). The APC and AAPC were presented with 95% confidence intervals (CIs). All p-values were 2-tailed, and statistical significance was set at p-value < 0.05. The statistical package SAS Enterprise Guide version 7.1 (SAS Institute Inc., Cary, NC, USA), R Studio version 3.3.3 (RStudio Inc., Boston, MA, USA), and Joinpoint Regression Program version 4.7.0.0 (National Cancer Institute, Bethesda, MD, USA) were used to perform the analyses in this study.

### Ethics statement

The Institutional Review Board of Chungbuk National University Hospital approved this study (CBNUH-2019-12-034) and waived the requirement of informed consent from study participants due to anonymity of the data obtained from the NHIS database. All research procedures followed the 1964 Declaration of Helsinki and were conducted in accordance with the relevant guidelines and regulations.

## RESULTS

### Baseline characteristics of the study population

Table 1 shows the baseline characteristics of the study population. The median age of subjects increased from 40.0 years in 2002 to 47.0 years in 2015. During the study period, the number of individuals newly prescribed opioids increased over time from 3 to 941 individuals. In particular, the number of new opioid users abruptly increased from 58 to 247 between 2008 and 2009.

### Trends in opioid prescriptions

Table 2 presents the age-standardized annual opioid prescription number (cases) per 10,000 registrants. The number of cases per 10,000 registrants increased from 0.07 (0.03 in male and 0.10 in female) in 2002 to 41.23 (40.96 in male and 41.15 in female) in 2015. The number of cases per 10,000 registrants tended to increase continuously in the elderly. Table 3 demonstrates the age-standardized amount of annual opioid prescriptions as MME per 10,000 registrants. The overall MME per 10,000 registrants increased from 15.06 (0.85 in male and 28.89 in female) in 2002 to 40,727.80 (45,263.38 in male and 35,603.54 in female) in 2015. Similar to the results presented in Table 2, increasing trends were observed in all subgroups.

### Joinpoint regression analysis for trends in opioid prescriptions

**Figure 1.**
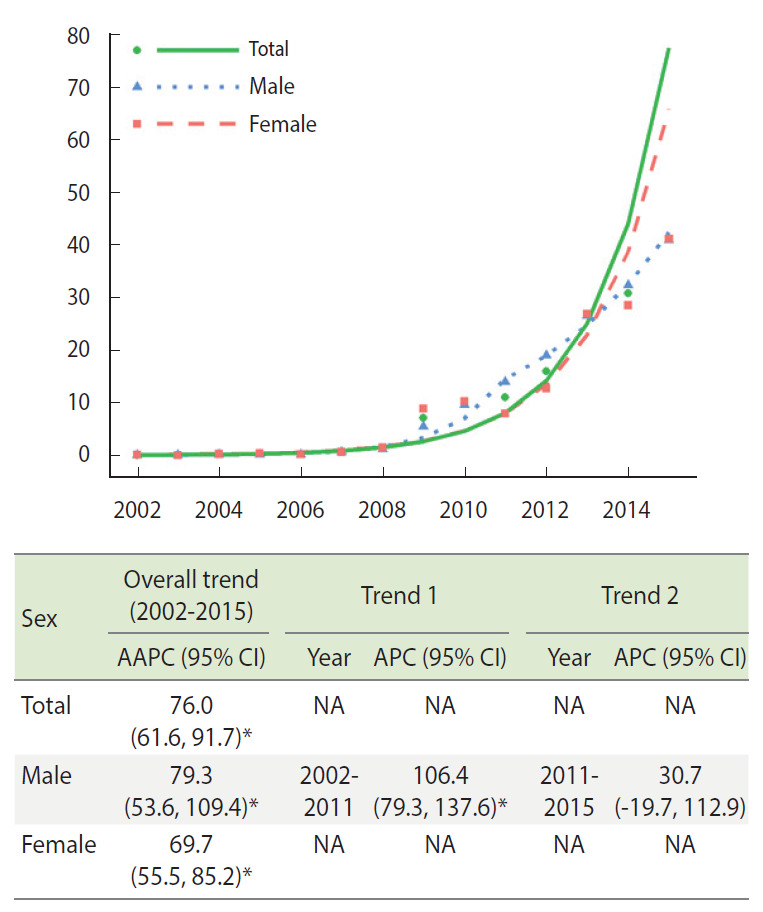
The age-standardized APC and AAPC of opioid prescriptions as cases per 10.000 registrants during 2002-2015 by sex. AAPC, average annual percentage change; APC, annual percentage change; CI, confidence interval; NA, not applicable. ^*^p<0.05.

Figure 1 demonstrates the trends in the age-standardized amount of annual opioid prescription as number (cases) per 10,000 registrants from the joinpoint analysis. The AAPC of the cases per 10,000 registrants in total, in male, and in female was 76.0% (95% CI, 61.6 to 91.7), 79.3% (95% CI, 53.6 to 109.4), and 69.7% (95% CI, 55.5 to 85.2), respectively. In males, a single joinpoint was estimated in 2011. The APCs were significant (106.4%; 95% CI, 79.3 to 137.6) between 2002 and 2011, but not between 2011 and 2015.

**Figure 2.**
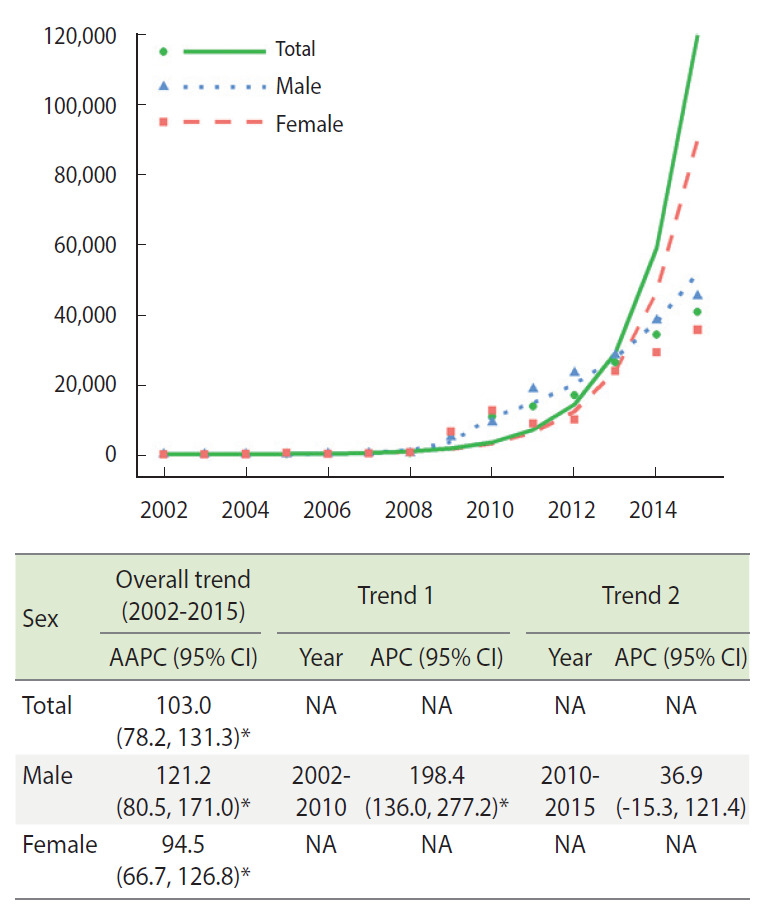
The age-standardized APC and AAPC of opioid prescriptions as milligram morphine equivalent per 10.000 registrants during 2002-2015 by sex. AAPC, average annual percentage change; APC, annual percentage change; CI, confidence interval; NA, not applicable. ^*^p<0.05.

Figure 2 presents the age-standardized trends in the amount of annual opioid prescriptions as the MME per 10,000 registrants. The overall trends represented as AAPC were 103.0% (95% CI, 78.2 to 131.3), 121.2% (95% CI, 80.5 to 171.0), and 94.5% (95% CI, 66.7 to 126.8) in total, in males, and in females, respectively. Similar to the results from Figure 1, a joinpoint was identified only for males in 2010. The APCs (95% CIs) were significant (198.4%; 95% CI, 136.0 to 277.2) between 2002 and 2010, but not between 2010 and 2015.

Further joinpoint regression analyses were performed by age group, medical institution, and opioid type (Supplementary Materials 1-4). Based on the annual number (cases) of opioid prescription per 10,000 registrants, the age groups of 30-39 years, 60-69 years, and 70-79 years had a single joinpoint in 2006, 2005, and 2004, respectively. By opioid type, fentanyl had a joinpoint in 2008, while hydromorphone had 2 joinpoints in 2007 and 2010. In terms of the annual amount (MME) of opioid prescription per 10,000 registrants, the age groups of 60-69 years and ≥ 70 years had a single joinpoint in 2005 and 2004, respectively. By opioid type, fentanyl had a joinpoint in 2005, while hydromorphone had 2 joinpoints, in 2007 and 2010.

## DISCUSSION

The number and amount of annual opioid prescriptions in Korea increased over the 14-year study period. The highest AAPCs were observed in the elderly population, patients in general hospitals, and patients receiving potent opioids such as fentanyl and hydromorphone.

The use of morphine is indicated for cancer pain treatment according to the WHO [16,17]. Morphine consumption has not increased in several countries since 2000, while increased use of other opioids, such as fentanyl and oxycodone, has been reported in many countries [18-22]. Although the quantity of morphine prescriptions has not increased greatly, the number of morphine prescriptions gradually increased in this study. Another Korean study also reported that chronic opioid use had increased since 2002 [23]. That study demonstrated that chronic use of strong opioids was positively associated with 5-year mortality, while weak opioid use was inversely related to mortality in Korea [23]. Although opioids are appropriately indicated for pain control for cancer patients, they should be used with caution.

Several factors appear to contribute to the increasing trends in overall prescriptions of opioids. First, the number of elderly patients increased. Age is closely associated with increased opioid use [24]. Studies from European countries between 2000 and 2010 reported that the utilization of strong opioids was highest among patients aged 66-80 years [21,25]. A study from Malaysia showed that opioid prescriptions increased with patient age (11% in the 40s, 19% in the 50s, and 47.28% in the 60-80s) [25]. Elderly people consume more opioids due to pain from multiple comorbidities. Second, the prevalence of diseases that cause pain, such as cancer and musculoskeletal diseases, increased [26]. Opioids are the most frequently used drugs to control cancer pain [2,27]. In 2017, there was a total of 232,255 new cancer cases in Korea [28]. Furthermore, the number of cancer survivors has been increasing due to the high incidence rate of cancers and the improved survival rates [28]. In addition, the Korean Ministry of Health and Welfare has supported more hospice and palliative care services since 2009 [29]. Because cancer is recorded with a special code, patients with cancer pay 5% of their total hospital costs, and the remaining hospital costs are paid by the NHIS. Thus, low out-ofpocket payments of cancer patients might result in more active pain management and increased prescriptions of pain medications such as opioids. In addition to the increased number of cancer patients as a factor influencing the upward trend in opioid prescriptions in Korea, opioid prescriptions have been gradually rising in patients with non-cancer diseases, including diseases that cause musculoskeletal pain [27]. Third, some new opioids were launched after 2000. Oxycodone was launched in the Korean market in 2001 and has taken, together with fentanyl, part of the market share of morphine. Hydromorphone was introduced to Korea in 2006, and since 2009, Korean national insurance has covered its use in treatment for cancer pain, which might have contributed to the dramatically increased prescriptions of hydromorphone. A sharp increase in use of fentanyl, oxycodone, and hydromorphone was identified. Higher growth rates for fentanyl, oxycodone, and hydromorphone use have been observed in other countries [18,20,21]. Fourth, awareness of active pain management has improved. The Korean Ministry of Health and Welfare launched pilot research for hospice and palliative care in 2005 and piloted a new payment system in 2009 [29]. The introduction and stabilization of hospice and palliative care have improved awareness and policies (e.g., insurance coverage) supporting more active pain management.

This study has several limitations. First, the data contained no clinical information on compliance with opioid medication prescriptions. Second, only strong opioids (morphine, fentanyl, oxycodone, and hydromorphone) were included in the analyses. Weak opioids, such as codeine and tramadol, are more commonly used but are not closely controlled by the Korean health authorities. Thus, it is difficult to estimate how many prescriptions of weak opioids were issued. On the contrary, because the Korean health authorities tightly regulate prescriptions of strong opioids, we can estimate usage trends relatively accurately. Third, the cohort from the Korean NHIS-NSC database was operated as a partially closed system. Only newborn infants were newly enrolled during the study period after the baseline. Thus, the accumulation of double-counted people each year might have contributed to the increasing pattern of opioid prescriptions. To minimize these errors, we calculated the total MME per year per 10,000 registrants after age-standardization. In addition, the number of patients who were newly prescribed opioids increased from 3 individuals in 2002 to 941 individuals in 2015. Fourth, since the clinical outcomes were not evaluated (e.g., whether patient symptoms such as pain were improved after administration of opioids), it was not possible to determine whether prescriptions of opioids directly led to an improvement in pain control. Fifth, we could not compare the increasing trends of opioid prescriptions with other pain killers such as acetaminophen or non-steroidal anti-inflammatory drugs as a control. Because these drugs are available without prescriptions in Korea, the exact amount of usage is infeasible to assess. If a comparison between opioids and other painkillers was possible, it would have been possible to identify trends in opioid usage more accurately. As an alternative method, we investigated age-standardized trends in opioid use, since changes in the age structure of the study population have a major impact on epidemiological studies.

There are several strengths of the present study. First, trends in both the number and amount of prescribed opioids over a period of 14 years were investigated using a nationally representative cohort. Second, trends in both the number and amount of annual opioid prescriptions per 10,000 registrants were estimated. Few previous reports have used a national patient sample, although an earlier study analyzed patterns of opioid prescriptions in Health Insurance Review and Assessment Service claims data in Korea [30]. Third, both short-term and long-term trends were examined. After the AAPCs for the entire study period were estimated, we calculated segmental short-term trends. This approach allowed us to infer factors influencing the increasing trends.

Opioid use disorder and overdose deaths currently pose a great threat to public health in the United States [31], while Europe does not appear to be facing an opioid crisis [32]. On the contrary, underuse has been more of a problem in Korea, as Korean culture disfavors opioid use and the Korean government strictly regulates strong opioids. However, this study reported that opioid prescriptions, calculated as the total prescription cases (numbers) and amount (MME), markedly increased over 14 years from 2002 to 2015 in Korea. These increasing patterns were prominent in the elderly population, patients treated at general hospitals, and patients prescribed potent opioids (fentanyl and hydromorphone). The finding that the legal use of opioids has increased suggests improved awareness of the importance of pain control over time. These increasing trends in opioid prescriptions and use in Korea will be very helpful in relieving and managing pain for patients with severe pain. However, policy-making to prevent misuse, dependence, addiction, and death from overuse is required.

## Figures and Tables

**Figure f3-epih-44-e2022029:**
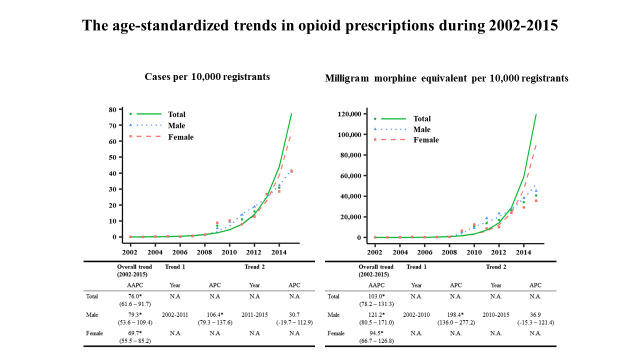


**Table 1.** Baseline characteristics of the study population

Characteristics		2002	2003	2004	2005	2006	2007	2008	2009	2010	2011	2012	2013	2014	2015
Total		725,224	739,052	752,581	765,674	778,544	791,680	799,645	808,031	817,608	827,503	837,231	846,676	855,723	864,702
Newly prescribed opioids		3	3	11	10	8	26	58	247	230	282	390	585	692	941
Age (yr)															
	Median±SD	40.0 (15.0)	41.0 (15.1)	41.0 (15.3)	42.0 (15.5)	43.0 (15.7)	43.0 (15.9)	44.0 (16.0)	44.0 (16.1)	45.0 (16.2)	45.0 (16.4)	46.0 (16.5)	46.0 (16.6)	47.0 (16.8)	47.0 (16.9)
	20-29	165,637	161,551	158,814	156,018	152,643	150,780	146,573	142,452	139,584	137,866	137,332	138,385	139,370	140,703
	30-39	184,223	184,735	183,333	182,252	180,735	176,416	174,077	172,308	170,090	168,247	165,070	160,918	158,132	155,271
	40-49	166,981	171,396	173,766	175,918	177,274	180,362	181,371	180,627	181,090	181,079	183,053	183,367	181,843	180,646
	50-59	95,871	101,144	109,172	115,781	122,083	128,355	135,945	145,291	154,323	159,761	164,474	168,493	170,538	172,342
	60-69	72,178	74,481	75,714	77,646	81,088	84,059	85,649	87,367	87,840	89,832	92,789	97,431	104,790	110,826
	≥70	40,334	45,745	51,782	58,059	64,721	71,708	76,030	79,986	84,681	90,718	94,513	98,082	101,050	104,914

SD, standard deviation.

**Table 2.** The age-standardized number of annual opioid prescriptions as cases per 10,000 registrants during 2002-2015

Variables		2002	2003	2004	2005	2006	2007	2008	2009	2010	2011	2012	2013	2014	2015
Prescriptions		0.07	0.04	0.22	0.32	0.18	0.64	1.33	7.15	9.99	11.05	16.01	26.81	30.78	41.23
Sex															
	Male	0.03	0.03	0.19	0.19	0.16	0.63	1.16	5.47	9.54	13.98	18.97	26.52	32.36	40.96
	Female	0.10	0.06	0.25	0.43	0.19	0.65	1.53	8.89	10.31	7.97	12.69	26.90	28.54	41.15
Age (yr)															
	20-29	0.00	0.00	0.03	0.00	0.00	0.00	0.02	0.00	0.00	0.56	0.75	0.54	0.83	0.83
	30-39	0.04	0.03	0.03	0.03	0.04	0.03	0.07	0.22	0.27	0.60	0.66	1.28	1.73	3.81
	40-49	0.00	0.00	0.06	0.00	0.03	0.14	0.09	0.47	0.76	1.45	2.29	3.85	5.08	6.58
	50-59	0.03	0.00	0.05	0.03	0.00	0.21	0.28	1.56	1.80	2.08	2.21	4.20	4.59	7.24
	60-69	0.00	0.00	0.03	0.14	0.06	0.12	0.51	1.92	3.66	3.29	5.68	9.04	9.46	11.52
	≥70	0.00	0.01	0.02	0.12	0.05	0.14	0.36	2.98	3.50	3.07	4.42	7.90	9.09	11.25
Medical institution type															
	General hospital	0.00	0.00	0.01	0.01	0.03	0.00	0.04	3.18	7.48	8.80	12.37	22.53	25.18	34.75
	Hospital	0.01	0.03	0.05	0.04	0.01	0.10	0.18	1.01	0.73	1.03	1.87	1.97	2.59	3.05
	Clinic	0.06	0.00	0.15	0.26	0.14	0.52	1.10	2.92	1.75	1.25	1.77	2.27	2.85	3.42
Opioid type															
	Fentanyl	0.00	0.00	0.01	0.05	0.01	0.10	0.95	4.31	4.13	4.13	4.95	7.70	8.26	10.51
	Oxycodone	0.04	0.02	0.10	0.21	0.14	0.25	0.25	2.35	5.28	5.55	8.66	14.60	16.78	24.82
	Hydromorphone	0.00	0.00	0.00	0.00	0.00	0.00	0.00	0.40	0.35	1.23	2.10	4.24	5.12	5.06
	Morphine	0.03	0.01	0.10	0.05	0.03	0.27	0.13	0.09	0.21	0.14	0.31	0.27	0.62	0.83

**Table 3.** The age-standardized number of annual opioid prescriptions as milligram morphine equivalent per 10,000 registrants during 2002-2015

Variables		2002	2003	2004	2005	2006	2007	2008	2009	2010	2011	2012	2013	2014	2015
Prescriptions		15.06	3.38	72.51	221.44	168.70	355.50	501.17	5,727.09	10,837.51	13,793.05	16,838.76	26,243.82	34,206.90	40,727.80
Sex															
	Male	0.85	4.22	62.07	35.70	210.66	373.86	402.55	4,974.07	9,090.91	18,734.48	23,341.95	28,278.94	38,256.23	45,263.38
	Female	28.89	2.60	83.48	411.01	128.66	345.85	603.53	6,521.57	12,603.26	8,809.25	10,014.66	23,780.26	29,163.46	35,603.54
Age (yr)															
	20-29	0.00	0.00	1.00	0.00	0.00	0.00	10.09	0.00	0.00	1,406.94	3,832.59	1,066.72	916.83	874.94
	30-39	5.37	2.47	12.93	5.01	20.02	22.67	16.26	477.24	221.50	497.18	621.75	1,636.54	3,489.28	4,120.55
	40-49	0.00	0.00	14.80	0.00	65.96	135.40	39.56	291.27	698.78	2,787.19	2,253.61	3,101.23	6,337.56	7,355.27
	50-59	9.69	0.00	34.73	4.51	0.00	35.67	68.12	1,864.63	1,750.89	2,367.10	1,964.12	4,620.35	4,801.05	7,055.79
	60-69	0.00	0.00	7.89	79.21	51.99	43.16	151.34	1,553.22	5,151.58	3,271.36	4,618.68	8,572.50	10,111.60	11,733.29
	≥70	0.00	0.91	1.16	132.71	30.73	118.60	215.80	1,540.73	3,014.76	3,463.28	3,548.01	7,246.48	8,550.58	9,587.96
Medical institution type															
	General hospital	0.00	0.00	1.07	3.35	21.36	0.00	8.12	3,782.35	9,705.51	12,691.00	15,066.66	24,349.74	30,267.67	38,362.58
	Hospital	0.41	2.47	34.73	1.77	0.86	68.13	72.84	514.83	331.91	567.10	1,100.18	946.11	1,390.79	1,254.08
	Clinic	14.65	0.00	36.62	216.33	146.47	287.36	412.90	1,395.31	796.60	534.97	671.90	865.01	1,232.51	1,098.99
Opioid type															
	Fentanyl	0.00	0.00	12.46	95.59	12.64	167.55	324.51	3,239.75	4,371.67	7,190.50	7,871.61	10,207.31	14,174.34	15,580.43
	Oxycodone	5.37	1.32	17.37	109.33	143.33	100.10	119.84	2,123.48	5,900.42	5,460.25	7,058.17	12,447.28	14,936.97	20,158.60
	Hydromorphone	0.00	0.00	0.00	0.00	0.00	0.00	0.00	303.84	391.83	1,083.57	1,814.88	3,387.20	4,516.84	4,529.21
	Morphine	9.69	2.06	42.68	16.51	12.72	87.84	56.83	60.01	173.60	58.74	94.06	202.01	578.77	459.56

## SUPPLEMENTARY MATERIALS

Supplementary Material 1. Annual percentage change and average annual percentage change of the amount of annual opioid prescription per 10,000 registrants during 2002-2015 by age group

Supplementary Material 2 Annual percentage change and average annual percentage change of the amount of annual opioid prescription per 10,000 registrants during 2002-2015 by opioid type

Supplementary Material 3 Age-standardized annual percentage change and average annual percentage change of the amount of annual opioid prescription cases per 10,000 registrants during 2002-2015

Supplementary Material 4 Age-standardized annual percentage change and average annual percentage change of the amount of annual opioid prescription as MME per 10,000 registrants during 2002-2015
