# Supplementary figures and images for: Recent trends in opioid prescriptions in Korea from 2002 to 2015 based on the Korean NHIS-NSC cohort
Source: Epidemiol Health. 2022 Feb 21;44:e2022029. doi: 10.4178/epih.e2022029 (PMC9117092; doi:10.4178/epih.e2022029)

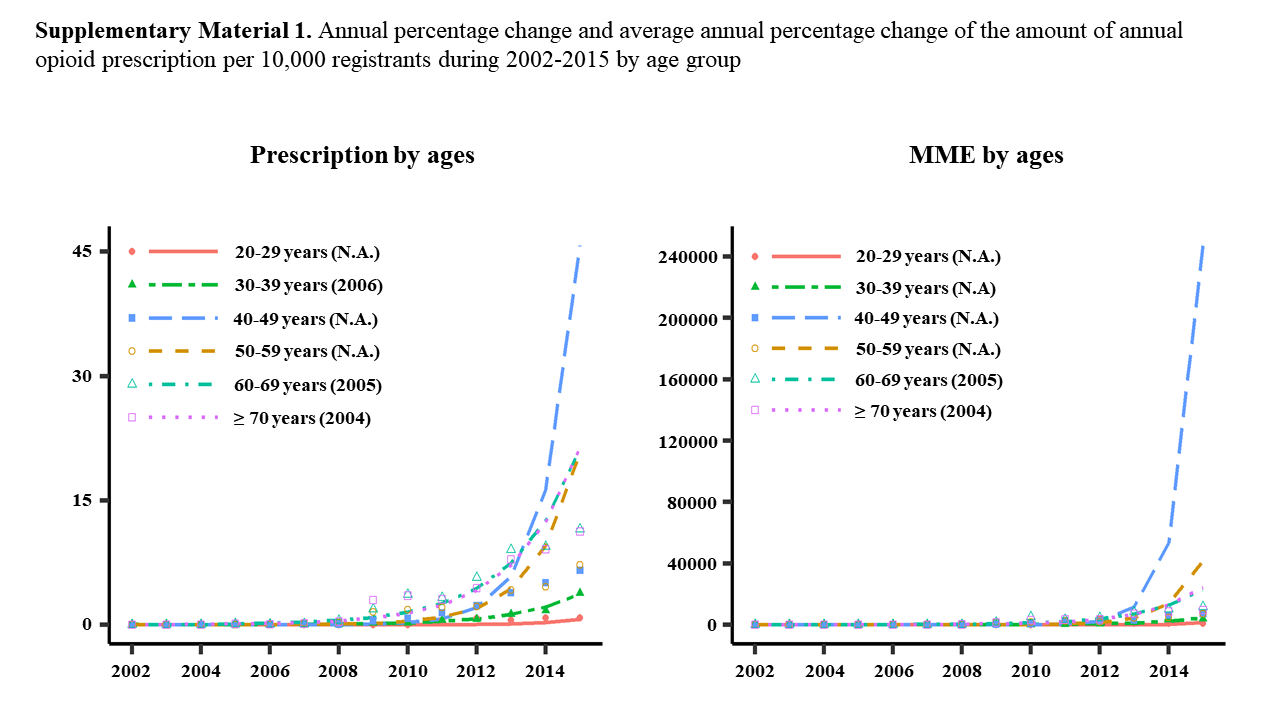

Supplement: Supplementary Material 1. — Annual percentage change and average annual percentage change of the amount of annual opioid prescription per 10,000 registrants during 2002-2015 by age group [file epih-44-e2022029-suppl1.tif]

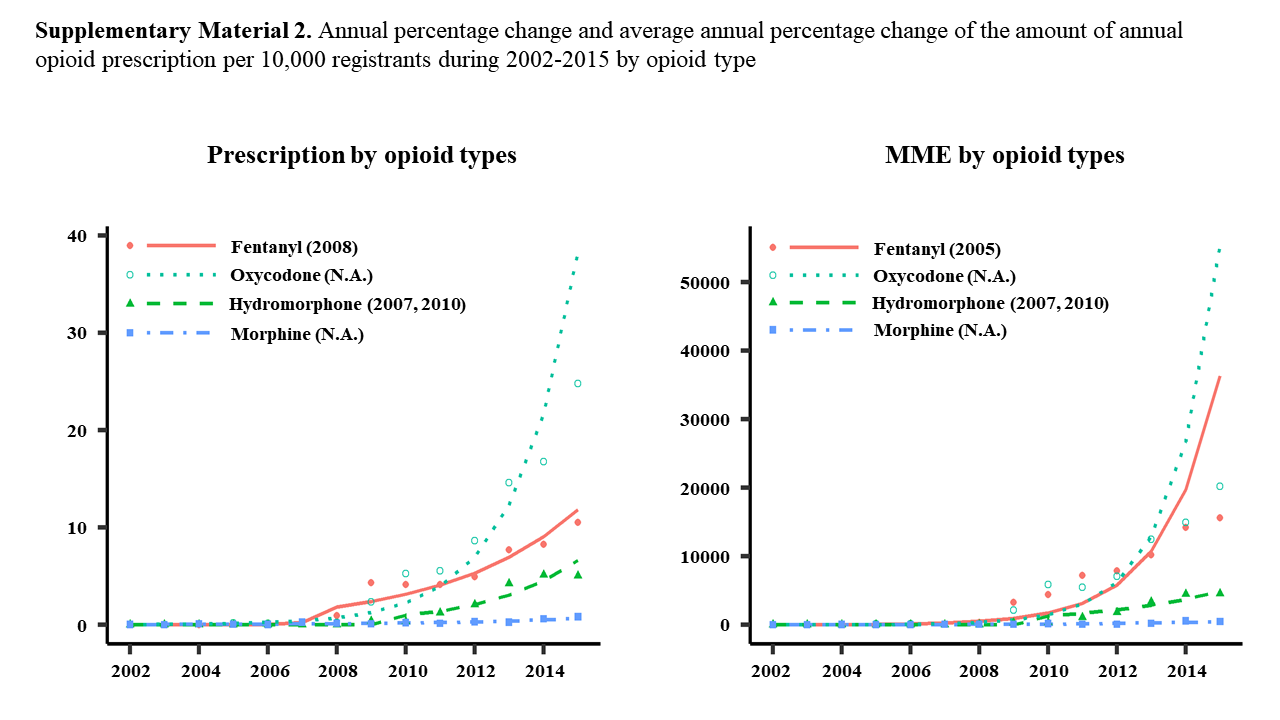

Supplement: Supplementary Material 2 — Annual percentage change and average annual percentage change of the amount of annual opioid prescription per 10,000 registrants during 2002-2015 by opioid type [file epih-44-e2022029-suppl2.tif]
