# Supplementary material for: Recent trends in opioid prescriptions in Korea from 2002 to 2015 based on the Korean NHIS-NSC cohort
Source: Epidemiol Health. 2022 Feb 21;44:e2022029. doi: 10.4178/epih.e2022029 (PMC9117092; doi:10.4178/epih.e2022029)
Supplement: Supplementary Material 3 — Age-standardized annual percentage change and average annual percentage change of the amount of annual opioid prescription cases per 10,000 registrants during 2002-2015 [file epih-44-e2022029-suppl3.docx]

**Supplementary Material 3**. Age-standardized annual percentage change and average annual percentage change of the amount of annual opioid prescription cases per 10,000 registrants during 2002-2015

|  | Overall trend (2002-2015) | Joinpoint analysis | | | | | |
| --- | --- | --- | --- | --- | --- | --- | --- |
|  |  | Trend 1 |  | Trend 2 |  | Trend 3 |  |
|  | AAPC, % (95% CI) | Year | APC, % (95% CI) | Year | APC, % (95% CI) | Year | APC, % (95% CI) |
| Prescription | 76.0 (61.6 – 91.7) ^*^ | N.A. | N.A. | N.A. | N.A. | N.A. | N.A. |
| Sex |  |  |  |  |  |  |  |
| Male | 79.3 (53.6 – 109.4) ^*^ | 2002 – 2011 | 106.4 (79.3 – 137.6) ^*^ | 2011 – 2015 | 30.7(-19.7 – 112.9) | N.A. | N.A. |
| Female | 69.7 (55.5 – 85.2) ^*^ | N.A. | N.A. | N.A. | N.A. | N.A. | N.A. |
| Age |  |  |  |  |  |  |  |
| 20~29 years | 153.6 (44.4 – 345.4) ^*^ | N.A. | N.A. | N.A. | N.A. | N.A. | N.A. |
| 30~39 years | 43.1 (31.3 – 55.9) ^*^ | 2002 – 2006 | -4.5(-27.1 – 25.2) | 2006 – 2015 | 71.2 (58.4 – 85.1) ^*^ | N.A. | N.A. |
| 40~49 years | 180.1 (92.8 – 306.9) ^*^ | N.A. | N.A. | N.A. | N.A. | N.A. | N.A. |
| 50~59 years | 117.8 (38.6 – 242.2) ^*^ | N.A. | N.A. | N.A. | N.A. | N.A. | N.A. |
| 60~69 years | 230.0 (108.6 – 421.9) ^*^ | 2002 – 2005 | 2968.8 (300.7 – 23401.9) ^*^ | 2005 – 2015 | 69.0 (23.1 – 132.0) ^*^ | N.A. | N.A. |
| ≥70 years | 183.0 (101.5 – 297.5) ^*^ | 2002 – 2004 | 4179.3 (292.6 – 46547.5) ^*^ | 2004 – 2015 | 72.7 (47.0 – 102.9) ^*^ | N.A. | N.A. |
| Institute type |  |  |  |  |  |  |  |
| General hospital | 235.4 (120.0 – 411.3) ^*^ | N.A. | N.A. | N.A. | N.A. | N.A. | N.A. |
| Hospital | 60.1 (43.6 – 78.5) ^*^ | N.A. | N.A. | N.A. | N.A. | N.A. | N.A. |
| Clinic | 71.0 (19.3 – 145.2) ^*^ | N.A. | N.A. | N.A. | N.A. | N.A. | N.A. |
| Opioid type |  |  |  |  |  |  |  |
| Fentanyl | 185.1 (83.3 – 343.5) ^*^ | 2002 – 2008 | 609.9 (215.2 – 1498.9) ^*^ | 2008 – 2015 | 30.5 (-31.3 – 147.9) | N.A. | N.A. |
| Oxycodone | 76.4 (61.8 – 92.4) ^*^ | N.A. | N.A. | N.A. | N.A. | N.A. | N.A. |
| Hydromorphone | 175.5 (140.4 – 215.6) ^*^ | 2002 – 2007 | -15.9 (-27.3 – -2.8) ^*^ | 2007 – 2010 | 5572.5 (2858.7 – 10775.4) ^*^ | 2010 – 2015 | 47.0 (27.1 – 70.0) ^*^ |
| Morphine | 29.3 (17.9 – 41.8) ^*^ | N.A. | N.A. | N.A. | N.A. | N.A. | N.A. |

*The APC or AAPC is significantly different from zero (p<0.05).

Abbreviations: MME, morphine milligram equivalent; AAPC, average annual percentage change; APC, annual percentage change.
