# Supplementary material for: Recent trends in opioid prescriptions in Korea from 2002 to 2015 based on the Korean NHIS-NSC cohort
Source: Epidemiol Health. 2022 Feb 21;44:e2022029. doi: 10.4178/epih.e2022029 (PMC9117092; doi:10.4178/epih.e2022029)
Supplement: Supplementary Material 4 — Age-standardized annual percentage change and average annual percentage change of the amount of annual opioid prescription as MME per 10,000 registrants during 2002-2015 [file epih-44-e2022029-suppl4.docx]

**Supplementary** **Material 4**. Age-standardized annual percentage change and average annual percentage change of the amount of annual opioid prescription as MME per 10,000 registrants during 2002-2015

|  | Overall trend (2002-2015) | Joinpoint analysis | | | | | |
| --- | --- | --- | --- | --- | --- | --- | --- |
|  |  | Trend 1 |  | Trend 2 |  | Trend 3 |  |
|  | AAPC, % (95% CI) | Year | APC, % (95% CI) | Year | APC, % (95% CI) | Year | APC, % (95% CI) |
| Prescription | 103.0 (78.2 – 131.3) ^*^ | N.A. | N.A. | N.A. | N.A. | N.A. | N.A. |
| Sex |  |  |  |  |  |  |  |
| Male | 121.2 (80.5 – 171.0) ^*^ | 2002 – 2010 | 198.4 (136.0 – 277.2) ^*^ | 2010 – 2015 | 36.9 (-15.3 – 121.4) | N.A. | N.A. |
| Female | 94.5 (66.7 – 126.8) ^*^ | N.A. | N.A. | N.A. | N.A. | N.A. | N.A. |
| Age |  |  |  |  |  |  |  |
| 20~29 years | 387.2 (92.3 – 1134.5) ^*^ | N.A. | N.A. | N.A. | N.A. | N.A. | N.A. |
| 30~39 years | 80.9 (62.3 – 101.6) ^*^ | N.A. | N.A. | N.A. | N.A. | N.A. | N.A. |
| 40~49 years | 362.4 (136.1 – 805.5) ^*^ | N.A. | N.A. | N.A. | N.A. | N.A. | N.A. |
| 50~59 years | 202.4 (42.9 – 539.8) ^*^ | N.A. | N.A. | N.A. | N.A. | N.A. | N.A. |
| 60~69 years | 492.3 (183.7 – 1136.3) ^*^ | 2002 – 2005 | 31556.9 (1107.1 – 830139.1) ^*^ | 2005 – 2015 | 79.5 (8.0 – 198.6) ^*^ | N.A. | N.A. |
| ≥70 years | 364.5 (226.0 – 561.8) ^*^ | 2002 – 2004 | 64279.0 (5237.8 – 776371.5) ^*^ | 2004 – 2015 | 89.5 (60.2 – 124.1) ^*^ | N.A. | N.A. |
| Institute type |  |  |  |  |  |  |  |
| General hospital | 450.8 (169.5 – 1025.7) ^*^ | N.A. | N.A. | N.A. | N.A. | N.A. | N.A. |
| Hospital | 88.3 (54.7 – 129.2) ^*^ | N.A. | N.A. | N.A. | N.A. | N.A. | N.A. |
| Clinic | 96.9 (9.4 – 254.2) ^*^ | N.A. | N.A. | N.A. | N.A. | N.A. | N.A. |
| Opioid type |  |  |  |  |  |  |  |
| Fentanyl | 512.1 (179.6 – 1240.0) ^*^ | 2002 – 2005 | 33544.1 (938.3 – 1090022.0) ^*^ | 2005 – 2015 | 84.0 (7.0 – 216.2) ^*^ | N.A. | N.A. |
| Oxycodone | 107.1 (79.9 – 138.4) ^*^ | N.A. | N.A. | N.A. | N.A. | N.A. | N.A. |
| Hydromorphone | 352.7 (303.6 – 407.9) ^*^ | 2002 – 2007 | -24.7 (-33.4 – -14.8) ^*^ | 2007 – 2010 | 71467.3 (41194.2 – 123933.7) ^*^ | 2010 – 2015 | 30.4 (15.3 – 47.5) ^*^ |
| Morphine | 38.5 (24.1 – 54.5) ^*^ | N.A. | N.A. | N.A. | N.A. | N.A. | N.A. |

*The APC or AAPC is significantly different from zero (p<0.05).

Abbreviations: MME, morphine milligram equivalent; AAPC, average annual percentage change; APC, annual percentage change.
